# Chronic hepatitis E complicated by chylous ascites in an HIV-infected patient with previous disseminated MAC infection: a case report

**DOI:** 10.1128/asmcr.00034-25

**Published:** 2025-05-23

**Authors:** Alex Tanoto Lim, Yunn Cheng Ng, Chiaw Yee Choy

**Affiliations:** 1 Department of Infectious Diseases, Tan Tock Seng Hospital 63703, Singapore, Singapore; 2 National Centre for Infectious Diseases, Singapore, Singapore; 3 Department of Gastroenterology and Hepatology, Tan Tock Seng Hospital 63703, Singapore, Singapore; Rush University Medical Center, Chicago, Illinois, USA

**Keywords:** ascites, chylous, atypical mycobacteria, immunocompromised host, hepatitis E virus

## Abstract

**Background:**

Chronic hepatitis E is a well-recognized phenomenon in immunocompromised hosts, most typically in the post-transplant population. However, the typical case presentation is usually that of transaminitis leading to rapidly progressive cirrhosis.

**Case Summary:**

In this case report, we discuss the case of a 59-year-old gentleman living with HIV with a history of treated disseminated *Mycobacterium avium* complex infection treated 8 years prior, who initially presented with asymptomatic deranged liver enzymes and positive hepatitis E virus (HEV) RNA and subsequent recurrent chylous ascites, with good clinical and biochemical response to treatment with ribavirin.

**Conclusion:**

This case, to the best of our knowledge, is the second case of HEV infection presenting with chylous ascites and the first in a HIV-infected patient.

## INTRODUCTION

Hepatitis E virus (HEV) is a positive‐sense single‐stranded RNA virus and a leading cause of acute viral hepatitis worldwide, with an estimated global seroprevalence of 12.47% (1). It is considered endemic in much of Asia and Africa and is transmitted mainly fecal-orally in localities with poor sanitation and lack of access to potable water (2). The infection is typically asymptomatic or self-limiting in the general population but may develop into chronic hepatitis and/or extrahepatic neurologic manifestations such as Guillain-Barre syndrome in immunocompromised hosts, particularly transplant recipients (3, 4).

However, chronic hepatitis E in people living with HIV remains uncommon (5). To date, there have only been 12 cases of chronic HEV infection reported in people living with HIV, with the overall prevalence of chronic HEV infection among this population being between 0% and 0.5%, and symptoms typically being those of persistently deranged transaminase levels and rapid progression to liver cirrhosis (6).

In this article, we describe a patient with HIV who presented with abnormal liver function tests and chylous ascites with detected HEV RNA 7 months post onset of abnormalities. He thereafter responded both clinically and biochemically to antiviral therapy for chronic HEV.

## CASE PRESENTATION

A 59-year-old gentleman first presented to a tertiary hospital in Singapore in 2014, presenting with newly diagnosed advanced HIV and disseminated *Mycobacterium avium* complex (MAC), with mycobacteremia, pulmonary disease as well as retroperitoneal lymphadenopathy. For his MAC infection, he was initiated on a combination antimycobacterial therapy with rifabutin 150 mg once daily, ethambutol 800 mg once daily, and clarithromycin 250 mg twice daily for 3 months, to which he developed drug-induced bicytopenia. Thereafter, rifabutin was stopped, and only ethambutol and clarithromycin were continued for a further 18 months before switching to clarithromycin prophylaxis.

In the intervening years, he had incomplete immune reconstitution, with absolute CD4 count remaining between 60 and 80 cells/μL despite virologic suppression. Clarithromycin prophylaxis was stopped in December 2018 after a discussion with the patient. He received antiretroviral therapy with dolutegravir, abacavir, and lamivudine and was on statin therapy for hyperlipidemia. He was not on any traditional preparations or supplements.

He was hepatitis A non-immune and had prior exposure to hepatitis B, with a reactive anti-hepatitis B core total antibody but undetectable hepatitis B viral load. He was not vaccinated against hepatitis E, and his HEV immune status was not previously screened.

In November 2023, the patient’s HIV viral load was 93 copies/mL, with a CD4 count of 77 cells/μL. We noted new liver function test derangement (AST 50 U/L; ALT 63 U/L). Prior to this, his serum aspartate aminotransferase (AST) and alanine transaminase (ALT) values between 2020 and April 2023 had been within normal range. He had no fever, abdominal pain, nausea, jaundice, or ascites. The patient had no history of intravenous drug use, recent tattoos, ingestion of shellfish or raw/undercooked meats, or travel out of Singapore. He opted to monitor his liver function results with statin cessation.

However, by April 2024, his liver function tests continued to worsen, and in June 2024, he developed first-onset gross ascites affecting his appetite and bilateral pedal edema. He was referred to our Gastroenterology and Hepatology Specialist Outpatient Clinic.

Subsequent evaluation demonstrated a positive hepatitis E viral load of 7,170,000 IU/mL. Autoimmune hepatitis, thyroid function, and other infective hepatitis (hepatitis A/B/C, cytomegalovirus, Epstein-Barr virus, herpes simplex virus, and syphilis) testing were negative.

Computer tomography (CT) abdomen showed that the liver was normal in contour and attenuation, though there was noted diffuse mural edema of the cecum. No portal/hepatic vein thrombosis was seen on imaging.

An elective abdominal paracentesis was arranged in mid-July 2024, which yielded turbid fluid with a high serum albumin-ascitic albumin gradient (SAAG), negative bacterial and mycobacterial cultures, and non-malignant cytology (Table 1).

**TABLE 1:** Ascitic fluid investigations^*a*^

	July 2024 (first paracentesis)	August 2024 (second paracentesis)	August 2024 (third paracentesis)
RBC (cells/μL)	118		166
NC (cells/μL)	179 (macrophages 72%; neutrophils 25%)		71 (macrophages 39%; lymphocytes 34%)
Protein (g/L)			<15
Albumin (g/L)	<15 (serum albumin 21)	<15 (serum albumin 19)	<15 (serum albumin 17)
Triglycerides (mmol/L)		1.9	1.7 (serum triglycerides 0.9)
AFB smear and culture	No growth at 8 weeks of incubation	No growth at 8 weeks of incubation	No growth at 8 weeks of incubation
Fluid aerobic and anaerobic culture	No growth after 5 days		No growth after 5 days
Cytology	No high-grade malignant cells seen	No high-grade malignant cells seen	No high-grade malignant cells seen

^*a*^ RBC, red blood cell count (cells/μL); NC, nucleated cell count (cells/μL). Grey shading denotes values not determined.

In August 2024, he required two more sessions of large-volume paracentesis for rapidly reaccumulating symptomatic ascites, draining out 4,300 mL of turbid fluid and 8,250 mL of fluid in the second and third paracentesis, respectively. These fluid samples were sent for bacterial and mycobacterium cultures and cytology at each paracentesis, all of which returned negative.

**Fig 1:**
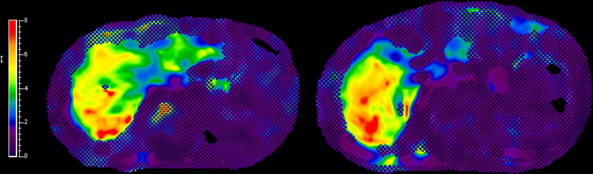
Magnetic resonance elastography August 2024 mean liver stiffness: 5.09 kPa (range: 0.96–8.85 kPa). <2.5 kPa = Normal | 2.5–3.0 kPa = Normal or inflammation. Readings that imply increased liver stiffness under appropriate clinical and laboratory findings: 3.0–3.5 kPa = Stage 1–2 fibrosis | 3.5–4 kPa = Stage 2–3 fibrosis | 4–5 kPa = Stage 3–4 fibrosis | >5 kPa =Stage 4 fibrosis).

**Fig 2:**
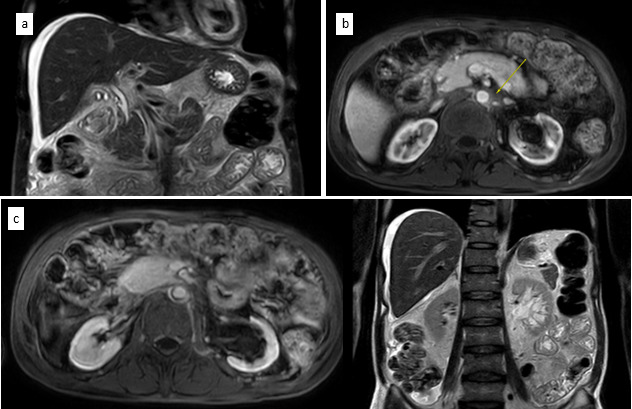
MRI abdomen images. (**a**) Mild ascites. Biliary tree not dilated. (**b**) Soft tissue thickening around left post-aortic region. (**c**) Left renal lymphangiectasia.

In view of his persistent mixed pattern deranged liver function tests with high SAAG ascitic fluid, an abdominal magnetic resonance imaging (MRI) with magnetic resonance cholangiopancreatography was arranged to evaluate for any obstruction of the biliary tree, and magnetic resonance elastography was done to evaluate for cirrhosis. Imaging showed a mean liver stiffness of 5.09 kPa, which could be compatible with stage 4 fibrosis, along with prominent upper abdominal nodes, mild perivascular soft tissue thickening in the left para-aortic region compatible with post-MAC treatment changes, and left renal lymphangiectasia, but no liver lesions or biliary obstruction (Fig. 1 and 2).

**Fig 3:**
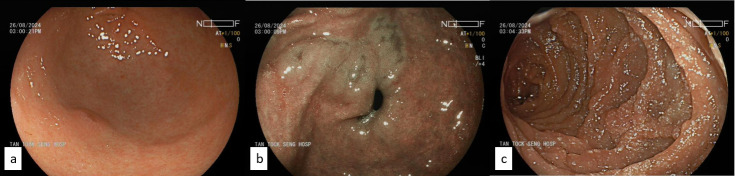
Duodenal lymphangiectasia seen on endoscopy in August 2024: (**a**) gastritis at the antrum, (**b**) intestinal metaplastic changes at the antrum on blue-light imaging, and (**c**) extensive duodenal lymphangiectasia.

He also underwent esophagogastroduodenoscopy and colonoscopy in August 2024 to evaluate the cecum thickening previously noted on CT abdomen. There were extensive gastric intestinal metaplastic changes and duodenal lymphangiectasia seen, which was confirmed on histology (Fig. 3).

During this period, the patient’s adherence to antiviral therapy had lapsed due to abdominal bloating from his ascites, and his HIV viral load increased to 4,490 copies/mL with a further CD4 count drop to 39 cells/μl. He had lost significant weight and muscle mass, with his weight decreasing from a baseline of 53 kg in May 2024 prior to ascites onset to 47.7 kg in August 2024 post-paracentesis. Given that his symptoms had preceded any changes in his CD4 count or viral load, this patient’s presentation was not in keeping with prior cases of chylous ascites associated with MAC-immune reconstitution syndrome (MAC-IRIS) (7). In view of the lack of alternative diagnoses and significant symptom burden with recurrent rapidly accumulating chylous ascites and malnutrition, a provisional diagnosis of chronic hepatitis E infection was made.

After discussing with the gastroenterology team the options of a liver biopsy for confirmatory diagnosis versus trial of ribavirin, the patient opted to start a trial of ribavirin treatment (dosing 200 mg twice daily as per dosing for chronic HEV infection in post-transplant host, renal-adjusted). He was reviewed by a dietitian and commenced on a minimal to low fat, high protein diet with medium chain triglyceride (MCT) (5 mL thrice daily) and oral nutritional supplementation (Resource Fruit 200 mL twice daily). He was advised to improve compliance with antiretroviral therapy.

**Fig 4:**
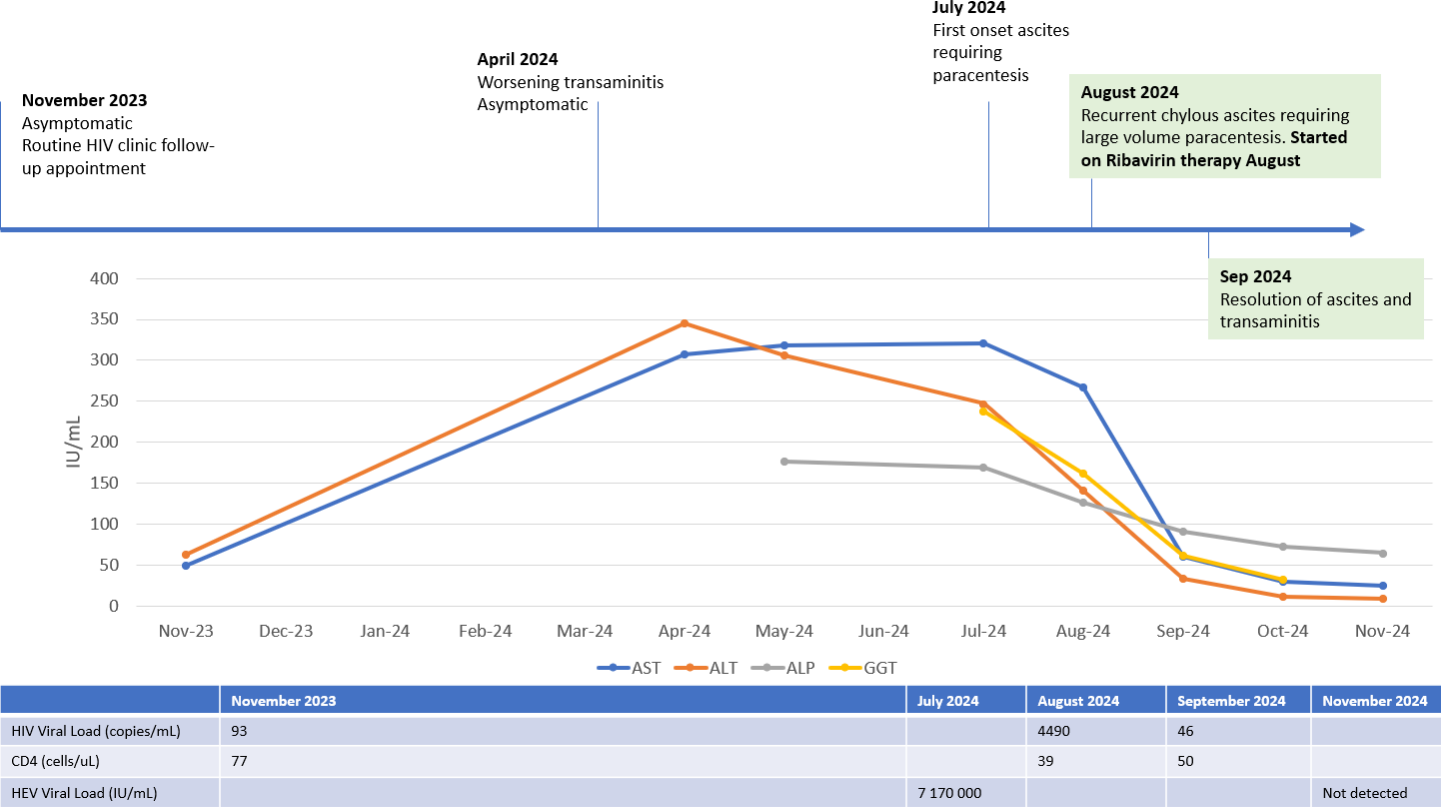
Overall clinical progress.

By 1 month of treatment with ribavirin, the patient’s ascites had resolved, and his liver function tests had almost normalized (Fig. 4). He completed a total of 12 weeks of ribavirin therapy, and his repeat HEV viral load was undetectable. With improved adherence to his antiretroviral therapy, his HIV viral load has decreased to 47 copies/mL, and CD4 has improved to 50 cells/μl. His MCT was stopped, and he was continued on a low-fat diet in view of hyperlipidemia. At 6 months after treatment, his repeat liver stiffness assessment via a one-dimensional ultrasound elastography (FibroScan) showed repeat liver stiffness assessment of 8.2 kPa compatible with stage 3 fibrosis. At 7 months post-completion of therapy, he continues to be well, with normal liver function tests and no recurrence of ascites.

## DISCUSSION

In this article, we describe a patient with HIV who presented with abnormal liver function tests and chylous ascites, with HEV viremia 7 months post onset of abnormalities. He thereafter responded both clinically and biochemically to antiviral therapy for chronic HEV. Given that Singapore is an endemic country for hepatitis E, direct molecular testing for HEV was pursued instead of serology (8).

Though the current clinical case definition of chronic hepatitis E requires documentation of persistent HEV viremia for more than 3 months duration (9), this was not pursued in this patient given the prolonged duration of liver function derangement prior to the first HEV viral load being sent and his rapid progression and debilitating nature of symptoms necessitating treatment initiation prior to diagnosis confirmation. However, the improvement in the patient’s symptoms and normalization of liver function tests coinciding with the initiation of HEV therapy in the absence of other management is supportive of the provisional diagnosis of chronic HEV infection.

Chylous ascites is not a typical presenting complaint associated with HEV, being more typically ascribed to damage to the lymphatic system from abdominal surgery, abdominal malignancy, or active mycobacterial infection (10). This contributed to our hesitancy in initiating anti-HEV antivirals immediately. Extrapolating from the incidence of lymphatic disruption during active mycobacterial infection/MAC-IRIS, it was hypothesized that residual scarring of the lymphatic system from the previous disseminated MAC infection led to permanent lymphatic dysfunction, also explaining the patient’s lymphangiectasia (11, 12). The inflammation caused by HEV infection could have led to the production of intraperitoneal fluid beyond the capacity of this dysfunctional system to drain. A similar case of HEV infection precipitating presentation with abnormal liver function tests and chylous ascites on a background of newly diagnosed intra-abdominal lymphoma has been described by Davern et al. (13).

An alternative hypothesis was that the patient had developed rapid onset cirrhosis due to HEV infection, given that the initial MR elastography demonstrated stage 4 fibrosis. However, as there were no other biochemical or radiological markers of cirrhosis, this may have been a false elevation of results due to ongoing liver inflammation at the time of imaging. While it is not possible to directly compare MR elastography and FibroScan results, it is promising that the FibroScan reading post-ribavirin therapy was only 8.2 kPa (> 12 kPa is suggestive of advanced fibrosis/cirrhosis) (14). We have the intention to repeat FibroScan in 1 year to monitor for further improvement.

Current treatment for chronic HEV infection in patients with HIV is unclear. Patients with CD4 counts >200 cells/ul appear to be able to spontaneously clear the virus after acute infection, whereas those with CD4 <200 cells/ul appear to develop persistent hepatitis, which only clears after CD4 recovery or antiviral therapy with ribavirin/pegylated interferon (15). This case shows promise with the use of 12 weeks of ribavirin to treat chronic HEV infection in an HIV patient (16, 17).

In conclusion, this case report raises awareness of the varying presentations of HEV infection in HIV patients with prior mycobacterial infection. Clinicians should be alert to the possibility of long-term sequelae of disseminated mycobacterial disease and consider treatment of HEV infection in such settings.
